# The Effects of Botulinum Toxin and Casting in Spastic Children With Cerebral Palsy: A Systematic Review and Meta-Analysis

**DOI:** 10.7759/cureus.36851

**Published:** 2023-03-29

**Authors:** Deepak Kumar, Rajan Kumar, Shiv K Mudgal, Priya Ranjan, Sanjay Kumar

**Affiliations:** 1 Department of Physical Medicine and Rehabilitation, All India Institute of Medical Sciences, Deoghar, Deoghar, IND; 2 Department of Pediatrics, All India Institute of Medical Sciences, Deoghar, Deoghar, IND; 3 College of Nursing, All India Institute of Medical Sciences, Deoghar, Deoghar, IND; 4 Department of Anesthesiology and Critical Care, All India Institute of Medical Sciences, Deoghar, Deoghar, IND

**Keywords:** meta-analysis, botulinum toxin type a, serial casting, spasticity, cerebral palsy

## Abstract

Cerebral palsy (CP) is a neurological disorder that affects muscle tone, movement, and motor skills in children. One of the most common symptoms of cerebral palsy is spasticity, which is characterised by involuntary muscle contractions and stiffness. Both botulinum toxin and casting have been used as standalone treatments for spasticity in cerebral palsy, but which is better is still unclear. The aim of the present meta-analysis was to compare the effects on spasticity of serial casting and/or botulinum toxin type A (BoNT-A) in conjunction with or as independent therapies. Studies up to February 2022 were identified in four separate databases. The inclusion criteria were randomised controlled trials (RCTs) that compared different therapies (Botulinum toxin A, or BoNT-A, and casting) and assessed spasticity improvement in children with spastic cerebral palsy who were younger than 18 years old and were published in English. With a 95% confidence interval (CI), the standardised mean difference (SMD) was utilised to calculate treatment effects. The Preferred Reporting Items for Systematic Reviews and Meta-Analyses (PRISMA) 2020 checklist was followed to undertake the current study. The search for relevant literature in four databases generated 147 results. After the abstract and full-text screening, five publications with a total of 190 cerebral palsy patients were included in this systematic review and meta-analysis. In patients with cerebral palsy, both methods - botulinum toxin and casting- apply globally; our systematic review tries to find out the most effective treatment between the two but does not show any significant difference in these methods. As we know, botulinum toxin is expensive, and the casting method is time-consuming and poorly accepted by patients. There is a need for an excellent study to examine the impact of casting and botulinum toxin type A.

## Introduction and background

The most frequent underlying condition resulting in motor impairment in children is cerebral palsy (CP) [1]. It is defined as 'a group of permanent disorders of posture and movement causing limitation of activity that ensues because of non-progressive disturbances that occurred during the developing foetal and infant brain'. Together with motor disturbances, it is associated with impairments of behaviour, cognition, perception, sensation, communication, epilepsy, and secondary musculoskeletal impairments [1-3]. Across the world, it affects 2.11 in every 1000 live births, with a slightly lower rate in the western world [4-5]. As it is a disorder of motor limitation, the focus of treatment by rehabilitative medicine is to improve mobility, and hence it has a significant role in enhancing the functional quality of life of children with cerebral palsy.

There are various classification systems for the different types of cerebral palsy. According to the 'Surveillance of Cerebral Palsy in Europe (SCPE) classification', the cerebral palsy can be classified into three major groups: spastic, dyskinetic, and ataxic types [6-8].

Spastic cerebral palsy, which accounts for about 85.8% of all diagnoses of cerebral palsy, is the most prevalent variety [9-10]. In the absence of relevant management, there will be contractures of muscles, ligaments, and tendons, which may have severe adverse outcomes on the functionality of children with cerebral palsy [11-12]. For instance, contracture of the calf muscle causes ankle flexion or equinus posture of the foot, which negatively affects standing, balancing, and walking. Contracture of the pelvic floor muscle results in hip flexion deformity [13-15]. Interventions are therefore concentrated on enhancing lower limb posture, which is expected to improve balance and gait. Several studies have shown that contractures, especially of the lower limbs, have a big effect on a child's ability to walk and do other important daily tasks [3]. To reduce negative impacts and increase functional outcomes, it is obvious that the best intervention options must be identified.

All the children with CP, according to their definition, have some sort of motor dysfunction. Clinical management of motor impairments like spasticity has either conservative management, invasive management, or a combination of both. Conservative strategies like physiotherapy, occupational therapy, orthoses, and casting have a central role in enhancing motor control [16]. Physical therapy helps kids learn posture, walking, toileting, and feeding in cerebral palsied kids, whereas occupational therapy seeks to improve function related to activities of daily living, work, and education [17]. Further, orthoses are devices that, with the help of external forces, attempt to improve the body's posture. Orthoses can be of the static type, which only supports target joints and prevents deformity, or the dynamic type, which not only helps joint alignment but also assists and stimulates movement [17]. Serial casting is another method that is employed to reduce spasticity in CP. When compared to a single fixed casting, serial casting is a technique that involves applying two or more successive fibreglass or plaster casts to a joint in order to increase the passive range of motion [3,18].

Invasive strategies are other commonly employed methods that mitigate spasticity and curb contracture development. These include intramuscular injections of botulinum toxin type A (BoNT-A), phenol, and alcohol and intrathecal injections of baclofen. With regard to botulinum toxin, when administered intramuscularly, it prevents the release of acetylcholine at the neuromuscular junction, resulting in selective chemodenervation for about two to three months, helping to reduce spasticity and ameliorating range of motion [16]. It can be combined with orthoses, serial casting, or intensive physical therapy to get the desired results [16,18]. In addition to the above-mentioned strategies, surgical interventions like selective dorsal rhizotomy or tendon lengthening procedures are also in vogue [16].

## Review

Methods

This study was performed using the 'Preferred Reporting Items for Systematic Reviews and Meta-Analyses (PRISMA) 2020 checklist, and the study was already registered in 'PROSPERO' with the number CRD42022372220 [19].

Search strategy

Four electronic databases - PubMed, Embase, Scopus, and Google scholar - were used to search for all English-language articles published from the beginning of the study until October 2022. The goal of the search strategy was to find out how cerebral palsy patients with spasticity responded to serial casting versus casting after botulinum toxin injections. The following keywords and MeSH terms were utilised in the study: Cerebral Palsy OR CP AND Serial Cast OR POP Cast OR casting AND Botulinum Toxin OR BoNT-A OR Botulinum toxin type A AND Spasticity OR increased tone. The reference lists of relevant trials were also examined in order to guarantee a full identification of additional possible publications. The search was restricted to studies involving humans only.

Eligibility criteria

The process of screening citations was sped up with the use of software called Zotero, which is a literature management programme. When the search results were imported into Zotero, duplicates were automatically eliminated on their own without any intervention from the user. After that, the titles and abstracts of the remaining publications were evaluated by two different writers: DK and SKM. This systematic analysis incorporated the complete versions of the publications that had been chosen to participate in those articles and satisfied the following inclusion criteria: (1) randomised controlled trials (RCT) on humans using a parallel or crossover design; (2) studies using serial casting and/or botulinum toxin type A (BoNT-A) in conjunction with or as independent therapies to compare their effects on spasticity; (3) children with stiffness in their upper and/or lower limbs; and (4) articles that are exclusively published in English. Studies were disqualified if any of the following conditions were met: (1) uncontrolled trials, (2) studies without full texts, and (3) research that lacked acceptable data reporting.

Data extraction

Two investigators did the data extraction on their own, based on the inclusion criteria. The following information was taken from the studies that were eligible: the first author's name, publication year, study’s design, the study group, the size of the sample, the length of treatment, the method of treatment, the baseline values, the endpoint values, or the net changes in spasticity. When the two investigators disagreed about whether or not a study was eligible, they discussed it or asked a third investigator for help.

Quality assessment

A trustworthy tool for evaluating the level of methodological rigour present in clinical trials is the 'Physiotherapy Evidence Database (PEDro)'. It is permissible to sum the scores acquired from the individual item ratings on the PEDro scale in order to produce a total score that may be treated as an interval-level measurement and submitted to parametric statistical analysis. Two investigators independently evaluated the quality of selected studies. The PEDro scale was developed by the Physiotherapy Evidence Database to rate the calibre of clinical research. The PEDro scale includes a questionnaire of ten scored yes-or-no questions about internal validity and statistical information. The scoring system is divided into three categories: great quality = 6-10, fair quality = 4-5, and bad quality = 3 [20]. In our study, PEDro out of 10 got more than or equal to 7 score. Thus, in our systematic analysis, all articles are good.

Statistical analysis

Using Review Manager Software, a meta-analysis was done (RevMan version 5.3.5, Copenhagen: The Nordic Cochrane Centre, The Cochrane Collaboration). We assessed the effects of treatment on spasticity by averaging the mean and standard deviation of endpoint values across the treatment and control groups. The pooled data were calculated using an inverse variance-weighted approach and displayed as a weighted mean difference (MD) with 95% confidence intervals (CI).

The I2 statistic was used to quantitatively measure heterogeneity. We considered I2 > 50% to be indicative of significant trial heterogeneity [21]. Where the studies were sufficiently heterogeneous, a random-effects model was employed to pool the data; otherwise, a fixed-effects model was used. On the basis of the Z-score of overall effects, the meta-analysis yielded statistically significant results at the 0.05 level. According to the duration criteria of this systematic review, a sensitivity analysis was conducted to determine whether the intervention is effective for short-term treatments.

Results

Search Results and Study Characteristics

**Figure 1:**
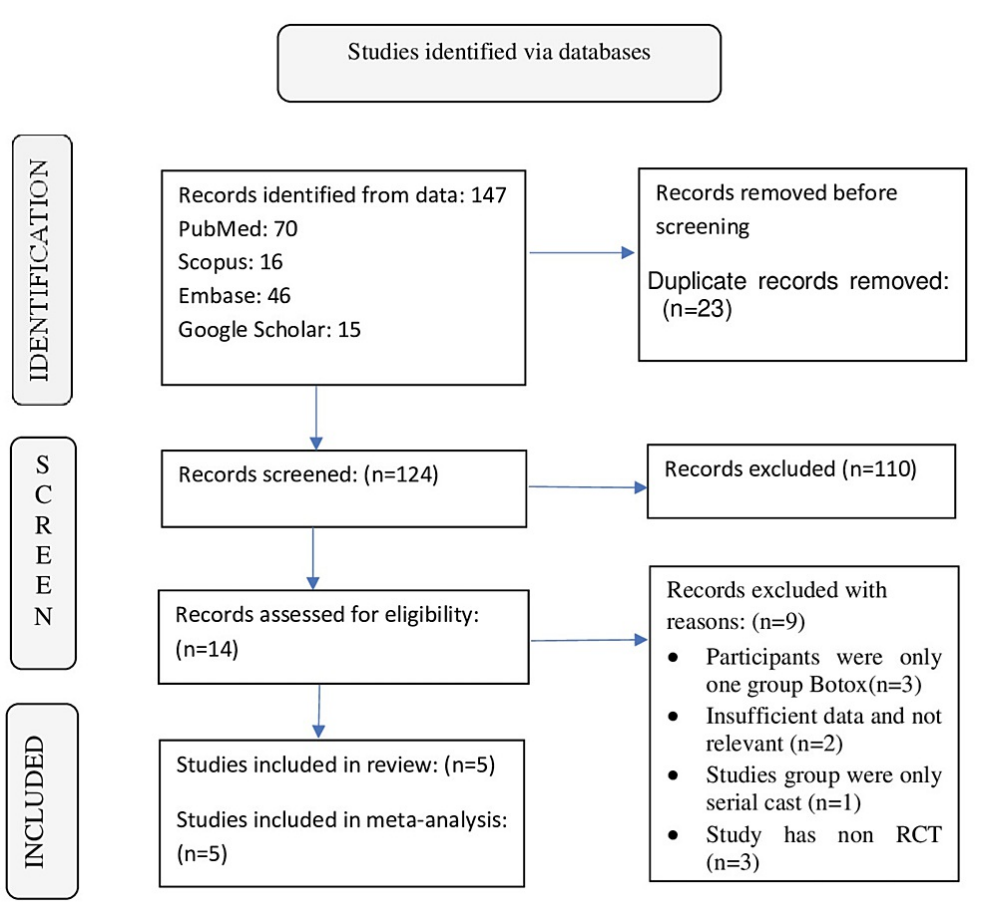
PRISMA flow diagram

Four databases were searched for relevant literature, yielding 147 items. This systematic review and meta-analysis included five papers with a total of 190 patients with cerebral palsy after the abstract and full-text screening. The literature screening process is described in the PRISMA flow chart (Figure 1).

The characteristics of the five papers are presented in Table 1 [22-26]. The sample sizes of the included studies varied from 10 to 70. Except for one, all of the included studies were single centres. The follow-up duration of the included studies ranges from 4 weeks to 48 weeks.

**Table 1:** Features of the included studies NR: not reported; RCT: randomized control trial

Author	Year	Design	Country	Sample size	Male cast	Female cast	Casting group	Male control	Female control	Control group
Dai and Demiryürek [22]	2017	RCT	Turkey	70	21	14	3.2(2.3)	19	16	3.4(2.1)
Ackman et al. [23]	2005	RCT	U.S.	25	6	7	6	6	6	5.9
Bottos et al. [24]	1999	RCT	Italy	10	NR	NR	6.4(2.7)	NR	NR	6.4(2.7)
Dursun et al. [25]	2021	RCT	Turkey	34	8	15	11.11(4.5)	7	4	9.0(4.5)
Dursun et al. [26]	2017	RCT	Turkey	51	21	13	75.5(36)	11	6	75.5(37.5)

Spasticity Level

**Figure 2:**
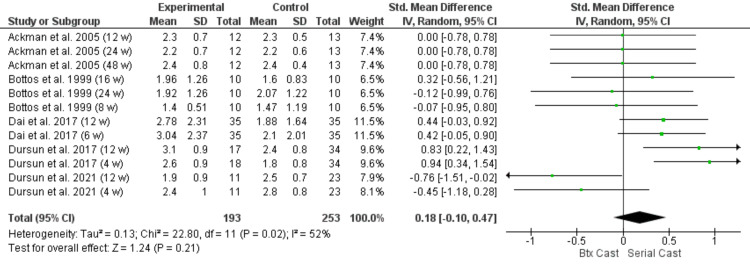
Comparison between botulinum toxin type A (Btx. Cast) vs. serial cast on the spasticity level Dai and Demiryürek [22]; Ackman et al. [23]; Bottos et al. [24]; Dursun et al. [25]; Dursun et al. [26]

Consolidated results from five studies (12 arms) showed no difference in decreasing the level of spasticity between the two groups, as evident by SMD 0.18, 95% CI −0.10 to 0.47; I2 52% P = 0.21 (Figure 2).

**Figure 3:**
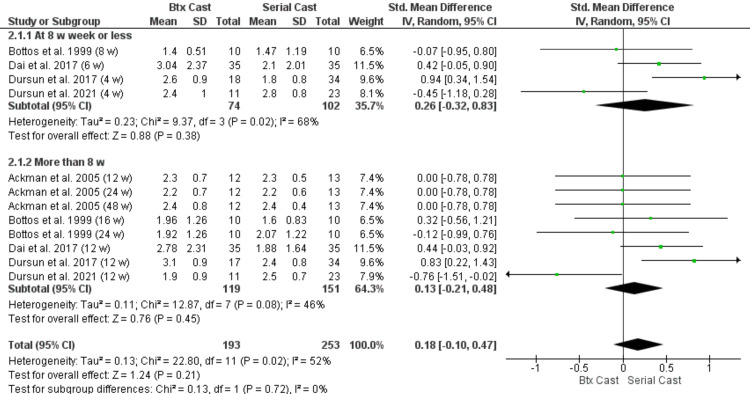
Comparison between botulinum toxin A (Btx. Cast) vs. serial cast on the spasticity level as per studies’ duration Dai and Demiryürek [22]; Ackman et al. [23]; Bottos et al. [24]; Dursun et al. [25]; Dursun et al. [26]

We conducted further analysis due to the significant level of variability. Spasticity at eight weeks or less and more than eight weeks were the study categories. The findings of spasticity level at eight weeks or less (SMD=0.26; CI −0.32; 0.83; I2=68%; p=0.38) and at more than eight weeks (SMD=0.13; CI −0.21;0.48; I2=46%; p=0.45) (Figure 3). This demonstrated that there was no difference in study time across the groups.

Discussion

CP with spasticity is the most prevalent kind. There are around 85.8% diagnoses of this type [27]. This is an important systematic review that compared the controversial treatments of serial casting versus botulinum toxin with a cast in spastic cerebral palsy. This review's objective was to add to the researchers' already extensive body of information. As far as we are aware, no recent study has summarised the findings of trials with greater methodological quality to determine the impact of serial casting on lower limb spasticity. However, no recent articles have performed a meta-analysis of pertinent results to enable more firm conclusions. But there are several kinds of treatment for spasticity, such as botulinum toxin, medicine, exercise therapy, splinting, and serial casting. The usefulness of serial casting as a supplement to pharmaceutical management has been the subject of discussion in a number of other systematic evaluations [1-2].

There are several controversial treatments for it, as even our included article shows the study by Durum et al. [26] has a significant positive result, but in the study by Durum et al. [25], botulinum toxin was a better option. There were also different results for immediate and delayed treatment outcomes. Barbara et al. claim that a single injection of botulinum toxin produces better results due to better compliance by families and clinicians. In one of the articles by Blagrove et al. [28], consistently, parents preferred botulinum toxin A and emphasised the discomforts of serial casting. The results of this one-year study show that BTX-A alone did not improve the parameters measured in this study. However, casting and Botulinum Toxin-A vs. casting were effective in the short-term and long-term treatment of spasticity in CP children. In Ackman et al. [23], serial casting has been suggested as a conservative treatment option to prevent gastrocnemius and soleus contractures. The study results of Dai and Demiryürek [22] suggest that putting a child in a cast after receiving botulinum toxin type A can make it work better for children with cerebral palsy.

We only talked about how well botulinum toxin and serial casting work to treat spasticity. Based on what this systematic review and meta-analysis found, serial casting (without BTX-A) may not have a big positive effect. The Modified Ashworth Score (MAS) was used to measure the amount of spastic hypertonia in the muscle by using a 6-point scale to measure resistance to passive stretching (from 0 for no increase in tone to 4 for rigidity in flexion or extension). A few of them also measure the Gross Motor Function Measure (GMFM), gait pattern, and Paediatric Evaluation of Disability Inventory (PEDI) as outcomes of the study. Blinding was not possible in most of the studies, so it can be considered a limitation of the study. This review led us to the conclusion that these advantages might have contributed to the reduction in spasticity and improvement in functional gait seen in some of the included trials. So, our systematic studies show no significant results for any of the particular treatments, BTX-A or serial casting. It is suggested to use artificial intelligence in managing spasticity in children with CP [29].

## Conclusions

In the end, these methods help reduce spasticity and increase both active and passive range of motion. In patients with cerebral palsy, both methods - botulinum toxin and casting - apply globally; our systematic review tries to find out the most effective treatment between the two but does not show any significant difference in these methods. As we know, botulinum toxin is expensive, and the casting method is time-consuming and not well accepted by patients. Research of the highest calibre is required to examine the effects of casting and botulinum toxin. Clinicians can create their own treatment strategies that are suitable and acceptable to them using the information from this systematic review.
